# Association between minor loading vein architecture and light- and CO_2_-saturated rates of photosynthetic oxygen evolution among *Arabidopsis thaliana* ecotypes from different latitudes

**DOI:** 10.3389/fpls.2013.00264

**Published:** 2013-07-22

**Authors:** Christopher M. Cohu, Onno Muller, Jared J. Stewart, Barbara Demmig-Adams, William W. Adams

**Affiliations:** Department of Ecology and Evolutionary Biology, University of ColoradoBoulder, CO, USA

**Keywords:** *Arabidopsis thaliana* ecotypes, leaf vasculature, light acclimation, phloem, photosynthesis, temperature acclimation, xylem

## Abstract

Through microscopic analysis of veins and assessment of light- and CO_2_-saturated rates of photosynthetic oxygen evolution, we investigated the relationship between minor loading vein anatomy and photosynthesis of mature leaves in three ecotypes of *Arabidopsis thaliana* grown under four different combinations of temperature and photon flux density (PFD). All three ecotypes exhibited greater numbers and cross-sectional area of phloem cells as well as higher photosynthesis rates in response to higher PFD and especially lower temperature. The Swedish ecotype exhibited the strongest response to these conditions, the Italian ecotype the weakest response, and the Col-0 ecotype exhibited an intermediate response. Among all three ecotypes, strong linear relationships were found between light- and CO_2_-saturated rates of photosynthetic oxygen evolution and the number and area of either sieve elements or of companion and phloem parenchyma cells in foliar minor loading veins, with the Swedish ecotype showing the highest number of cells in minor loading veins (and largest minor veins) coupled with unprecedented high rates of photosynthesis. Linear, albeit less significant, relationships were also observed between number and cross-sectional area of tracheids per minor loading vein versus light- and CO_2_-saturated rates of photosynthetic oxygen evolution. We suggest that sugar distribution infrastructure in the phloem is co-regulated with other features that set the upper limit for photosynthesis. The apparent genetic differences among *Arabidopsis *ecotypes should allow for future identification of the gene(s) involved in augmenting sugar-loading and -transporting phloem cells and maximal rates of photosynthesis.

## INTRODUCTION

Attempts to increase photosynthetic rate through overexpression of key components of the photosynthetic process have met with surprisingly little success (Micallef et al., 1995; Miyagawa et al., 2001; Lefebvre et al., 2005; Feng et al., 2007a, b; Kebeish et al., 2007; Rosenthal et al., 2011). If, however, any limitations were to exist to the *export* of products of photosynthesis from the leaf to the rest of the plant, one should, in fact, not expect increases in photosynthesis rate from overexpression of photosynthetic genes. In such an event, the well-known feedback inhibition by accumulated products of photosynthesis, via repression of photosynthetic genes (Krapp and Stitt, 1995; Paul and Foyer, 2001) and, possibly, sucrose transporter genes in apoplastic loaders (Vaughn et al., 2002), would be expected to counteract or abolish effects of overexpression of photosynthetic genes. To address the possible involvement of bottlenecks associated with transport processes, we characterized the foliar vascular system in several ecotypes of the plant model *Arabidopsis thaliana*.

The plant vascular system is composed of xylem (responsible for transport of water, nutrients, and other substances from the roots to the rest of the plant) and phloem (responsible for transport of sugars and other substances from sources, such as mature leaves, to the plant’s sinks that utilize and store products of photosynthesis). A correlation between photosynthesis and xylem hydraulic conductivity (Hubbard et al., 2001; Brodribb et al., 2005; Nardini et al., 2005; Sack and Holbrook, 2006) has thus far been the focus of studies on the relationship between photosynthesis and leaf venation (Boyce et al., 2009; Beerling and Franks, 2010; Brodribb and Feild, 2010; Brodribb et al., 2010; McKown et al., 2010; Blonder et al., 2011; Walls, 2011). In contrast, little attention has been given to a possible relationship between sugar export via the phloem and photosynthesis (Adams et al., 2013). In the studies relating photosynthesis to xylem hydraulic conductivity, photosynthesis was assessed as CO_2_ exchange between the leaf and the atmosphere. These measurements of the leaf’s CO_2_ fixation rates also reflect barriers to CO_2_ movement from the atmosphere to the carboxylation sites in the chloroplasts (cuticular, stomatal, mesophyll, cell wall, and chloroplast envelope resistances; Boyer et al., 1997; Tuzet et al., 2003; Tholen and Zhu, 2011; Flexas et al., 2012), and do not measure the leaf’s intrinsic maximal capacity for photosynthesis *per se*. In contrast, all resistances to CO_2_ diffusion to the sites of carboxylation are eliminated (via saturation of the leaf with 5% CO_2_) through use of the leaf disc oxygen electrode (Delieu and Walker, 1981) that allows determination of the intrinsic maximal rate of photosynthesis. In order to reveal any correlations between plant vascular features associated with sugar or water flux capacity and the maximal intrinsic potential for photosynthesis, light- and CO_2_-saturated photosynthetic oxygen evolution unhindered by resistances to the movement of CO_2_ needs to be assessed. We conducted a thorough examination of the leaves of three *A. thaliana *ecotypes grown under multiple environmental conditions to determine the relationship between phloem or xylem structure and the light- and CO_2_-saturated rates of photosynthetic oxygen evolution in this species. We grew all three *A. thaliana* lines under several photon flux densities (PFD) and temperatures, resulting in leaves with a wide range of vascular anatomical features (Cohu et al., 2013) and light- and CO_2_-saturated rates of photosynthetic oxygen evolution.

## MATERIALS AND METHODS

### *Arabidopsis thaliana* LINES

Three different ecotypes of *A. thaliana* (L.) Heynhold were investigated. Two were obtained from populations growing in north-central Sweden and central Italy, i.e., from the latitudinal extremes of this species’ natural geographic range ( Ågren and Schemske, 2012). The third was the wild-type Columbia (Col-0) line procured from The Arabidopsis Information Resource collection^1^.

### GROWTH CONDITIONS

Plants were grown from seed under controlled growth-chamber conditions (leaf temperature of 24–26°C day/20°C night resulting from air temperature 25°C day/20°C night, or leaf temperature of 12–16°C day/12.5°C night resulting from air temperatures of 8°C day/12.5°C night; elevated leaf temperature above air temperature due to daytime radiant heat gain), 9 h photoperiod (15 h dark) of 400 or 1000 μmol photons m^-^^2^s^-^^1^, and fertilized with nutrients every other day. Plants grown under conditions with an average daytime leaf temperature of 14°C (8°C air temperature during photoperiod) were germinated at an air temperature of 25°C until cotyledons emerged, then transferred to an air temperature of 15°C for 1 week before transfer to an air temperature of 8°C. Only fully expanded mature leaves of non-flowering plants at a similar stage of development (6–8 weeks old, with the plants subject to the two-step transfer to low temperature exhibiting a slightly offset development; see Cohu et al., 2013 for additional detail) that emerged under final growth conditions were characterized. Leaf temperatures were determined with a fine thermocouple thermometer (Wescor TH-65 meter, Logan, UT, USA) appressed to the lower surface of the leaves with porous (Transpore, 3M) tape.

### PHOTOSYNTHESIS AND VEIN MEASUREMENTS

**FIGURE 1:**
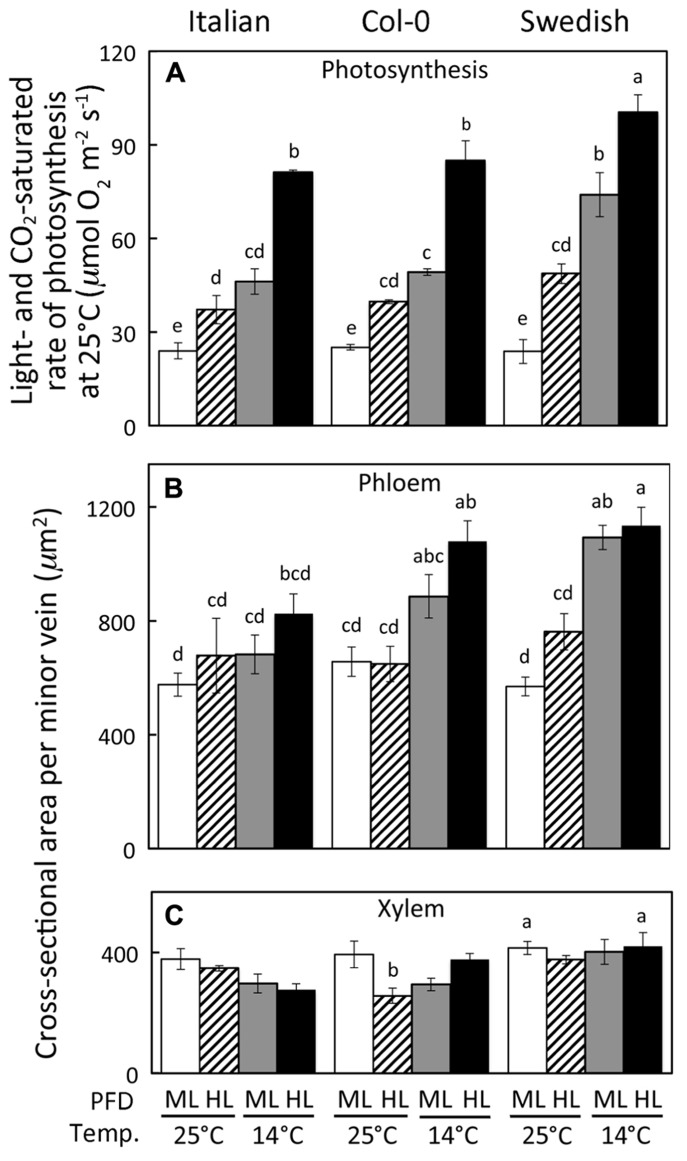
**(A)** Light- and CO_2_-saturated rate of photosynthetic oxygen evolution, (B) minor loading vein phloem tissue cross-sectional area, and **(C)** minor vein xylem tissue cross-sectional area of *A. thaliana *Italian, Swedish, and wild-type Col-0 ecotypes grown under controlled temperature (mean leaf temperature in °C) and PFD (ML = 400 and HL = 1000 μmol photons m^-^^2^ s^-^^1^) conditions. Actual daytime leaf temperatures varied between 24 and 26°C or between 12 and 16°C. Mean ± standard deviation shown for light- and CO_2_-saturated rate of photosynthetic oxygen evolution **(A)** and mean ± standard error of the mean shown for leaf vascular features **(B,C)** (*n* = 4 plants); statistically significant differences indicated with lower case letters (*P* < 0.05).

Measurements of light- and CO_2_-saturated rates of photosynthetic oxygen evolution at 25 or 12.5°C and leaf vein density, as well as leaf tissue embedding in Spurr resin were conducted as previously described (Amiard et al., 2005). Leaf minor loading veins (third- and fourth-order veins) consisted of phloem tissue with 14 or fewer sieve elements per vein while maintaining a greater than 50% phloem to vein cross-sectional area as established for *A. thaliana* by Cohu et al. (2013). Vein cross-sectional areas were determined by Image-J^2^(Rasband W.S., ImageJ, U.S. National Institute of Health, Bethesda, MD, USA, 1997–2012). Phloem and xylem parameters were quantified from 7–10 vein cross-sections per plant. Comparison of mean values (All pairs, Tukey HSD for **Figure 1**, and *t*-test for **Figure 3**), correlation coefficient, and level of significance (ANOVA) were determined using JMP statistical software (SAS Institute, Cary, NC, USA).

**FIGURE 2:**
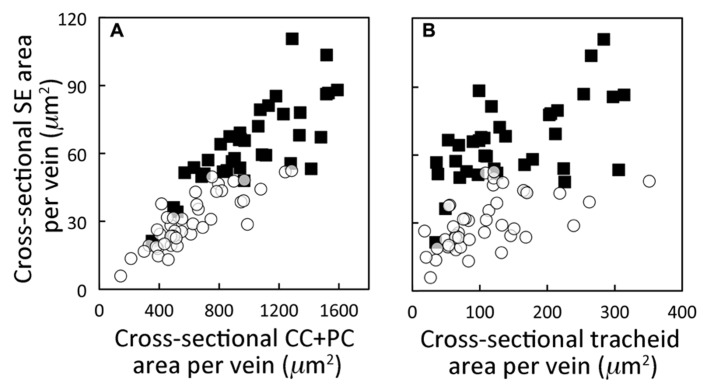
**Relationship between the cross-sectional area of the sieve elements (SE) versus (A) the cross-sectional area of the companion and phloem parenchyma cells immediately adjacent to those sieve elements and **(B)** the cross-sectional area of the tracheids in foliar minor loading veins of *A. thaliana *Italian (open circles) and Swedish (black squares) ecotypes grown in controlled temperature (12–16^°^C daytime leaf temperature) and moderate PFD (400 μmol photons m^**–2**^ s^**–1**^) conditions.** Area of sieve elements and area of tracheids is the sum of all sieve elements and tracheids, respectively, within a minor loading vein. The scatterplots represent individual foliar vein measurements from four plants per ecotype with 7–10 minor loading veins characterized per plant. Data points from the Swedish ecotype in **(B)** are from **Figure 5A in Cohu et al. (2013)**.

**FIGURE 3:**
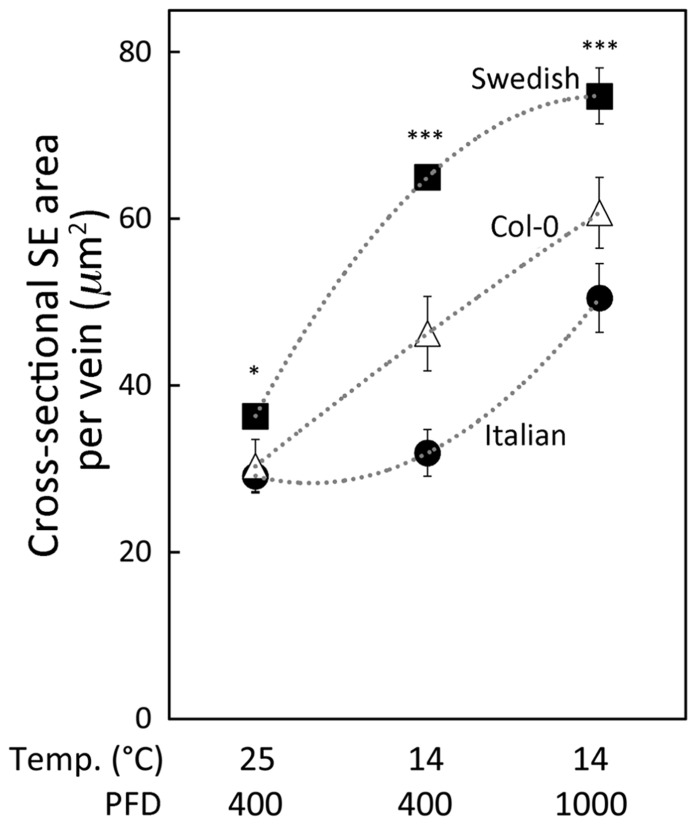
**Relationship between the cross-sectional area of sieve elements (SE) per minor loading vein and growth condition for three *A. thaliana *ecotypes (Italian ecotype, black circles; Col-0, white triangles; Swedish ecotype, black squares).** *Arabidopsis thaliana *ecotypes were grown in controlled temperature (mean daytime leaf temperature indicated in °C) and light (moderate PFD at 400, and high PFD at 1000 μmol photons m^-^^2^ s^-^^1^) conditions. Actual daytime leaf temperatures varied between 24 and 26°C or between 12 and 16°C. Mean ± standard error of the mean shown (*n* = 4 plants), and statistically significant differences between Swedish and Italian ecotypes for each growth condition indicated with an asterisk (*P < 0.05; ***P < 0.001).

## RESULTS

When the present study was initially undertaken, it was unknown which feature(s) of leaf vasculature, if any, may be adjusted in response to the environment. An extensive and comprehensive characterization of many different aspects of phloem anatomy was thus initially conducted (see Cohu et al., 2013). Those characteristics of the phloem (and xylem) that exhibited the greatest co-variation in response to the environment were explored here with respect to their relationship with photosynthesis.

Growth under higher PFD and lower temperature resulted in greater rates of light- and CO_2_-saturated photosynthetic oxygen evolution (**Figure 1A**) as well as greater cross-sectional areas of the phloem portion (**Figure 1B**) of foliar minor loading veins (veins active in loading sugars from the leaf’s photosynthetic mesophyll cells into sugar-transporting sieve elements; see Cohu et al., 2013) in the Italian, Col-0, and Swedish ecotypes. In addition to sieve elements, the phloem contains sieve-element-associated companion and parenchyma cells (Knoblauch and Oparka, 2012) in a ratio to sieve elements that is fairly constant among minor loading veins (**Table 1**; see also Amiard et al., 2007). Moreover, the Swedish ecotype exhibited significantly higher levels of both light- and CO_2_-saturated rates of photosynthetic oxygen evolution and phloem area than the Italian ecotype and the Col-0 line (**Figures 1A,B**) when grown under cool temperatures (14°C leaf temperature) at either moderate (400 μmol photons m^-^^2^ s^-^^1^) or high (1000 μmol photons m^-^^2^ s^-^^1^) PFD. In contrast to the phloem, the total cross-sectional area of the portion of minor loading veins occupied by the xylem tissue did not vary consistently among growth conditions or ecotypes over the range of growing conditions explored here (**Figure 1C**).

**Table 1:** Companion and phloem parenchyma cell (CC + PC) number per associated sieve element (SE) number in minor loading veins and vein density (vein length per leaf area; mm mm ^-2^) from leaves of Italian, Col-0, and Swedish ecotypes of *A. thaliana *grown under different controlled temperature (^°^C daytime leaf temperature) and photon flux density (PFD in μmol photons m^-2^s^-1^) conditions.

Ecotype	Growth conditions		CC + PC number/SE number	Vein density(mm mm^-2^)
	Temperature (°C)	PFD (μmol photons m^-2^s^-1^)
Italian	25	400	3.1 ± 0.07^ab^	2.84 ± 0.07 n.s.
	25	1000	2.97 ± 0.07^ab^	2.76 ± 0.31 n.s.
	14	400	3.11 ± 0.03^ab^	2.73 ± 0.29 n.s.
	14	1000	2.97 ± 0.08^ab^	2.93 ± 0.19 n.s.
Col-0	25	400	3.15 ± 0.11^ab^	2.88 ± 0.26 n.s.
	25	1000	3.07 ± 0.04^ab^	2.95 ± 0.13 n.s.
	14	400	3.25 ± 0.03^a^	2.45 ± 0.19 n.s.
	14	1000	3.11 ± 0.04^ab^	2.74 ± 0.18 n.s.
Swedish	25	400	3.00 ± 0.05^ab^	2.65 ± 0.19 n.s.
	25	1000	2.83 ± 0.10^b^	2.91 ± 0.28 n.s.
	14	400	3.14 ± 0.07^ab^	2.60 ± 0.23 n.s.
	14	1000	2.88 ± 0.03^b^	2.94 ± 0.32 n.s.

*Mean ± standard error of the mean (*n* = 4 plants). Statistically significant differences indicated with lower case letters (*P* < 0.05), n.s., not statistically different.*

Since the Italian and Swedish ecotypes exhibited the greatest differences in both light- and CO_2_-saturated rates of photosynthetic oxygen evolution and phloem cell area, these two ecotypes were compared for differences in the cross-sectional area of various cell types in individual foliar minor loading veins for leaves grown under cool temperatures and moderate PFD. **Figure 2A** shows that total cross-sectional area of sieve elements per vein increased in proportion with the total cross-sectional area of those phloem cells (companion cells, CC; and phloem parenchyma cells, PC) sharing a surface with sieve elements over a range of minor loading vein sizes. Furthermore, there was a high degree of segregation between data points for the Swedish ecotype (with larger cross-sectional sieve element and CC + PC areas) versus the Italian ecotype. Moreover, plotting of the combined cross-sectional area of all sieve elements in a minor loading vein versus the combined cross-sectional area of all water-transporting tracheids in a minor loading vein (**Figure 2B**) revealed an enhanced emphasis on sugar transport in the Swedish ecotype compared to the Italian ecotype. There was, again, almost complete segregation between data points for the Swedish versus the Italian ecotype, with a consistently larger cross-sectional area of the minor loading veins consisting of sieve elements versus tracheids in the Swedish ecotype (**Figure 2B**).

A greater responsiveness of sieve element size to a combination of cool temperature and higher PFD in the Swedish versus the Italian ecotype is illustrated in **Figure 3**. The cross-sectional area of minor loading veins occupied by sieve elements was modestly, albeit significantly, greater in the Swedish ecotype compared to the Italian ecotype when both ecotypes were grown under moderate PFD at 25°C (**Figure 3**, set of mean values to the left). Growth under the same moderate PFD but cooler temperature (14°C; **Figure 3**, middle set of mean values) did not result in increased sieve element area per vein in the ecotype from Italy. In contrast, in the Swedish ecotype sieve element area per vein was significantly greater under moderate PFD in plants grown at 14°C compared to 25°C, and more than twice that of the Italian ecotype when both ecotypes were grown at 14°C in moderate PFD (**Figure 3**, middle set of mean values). All three ecotypes exhibited significant additional increases in sieve element area when grown under high PFD at 14°C, with sieve element area per vein still significantly greater in the Swedish versus the Italian ecotype (**Figure 3**, set of mean values to the right). Total cross-sectional area of sieve elements per minor loading vein of the Col-0 line of *A. thaliana* exhibited intermediate values relative to the Italian and Swedish ecotypes, with significantly greater areas under cool temperature and increased growth light (**Figure 3**).

**FIGURE 4:**
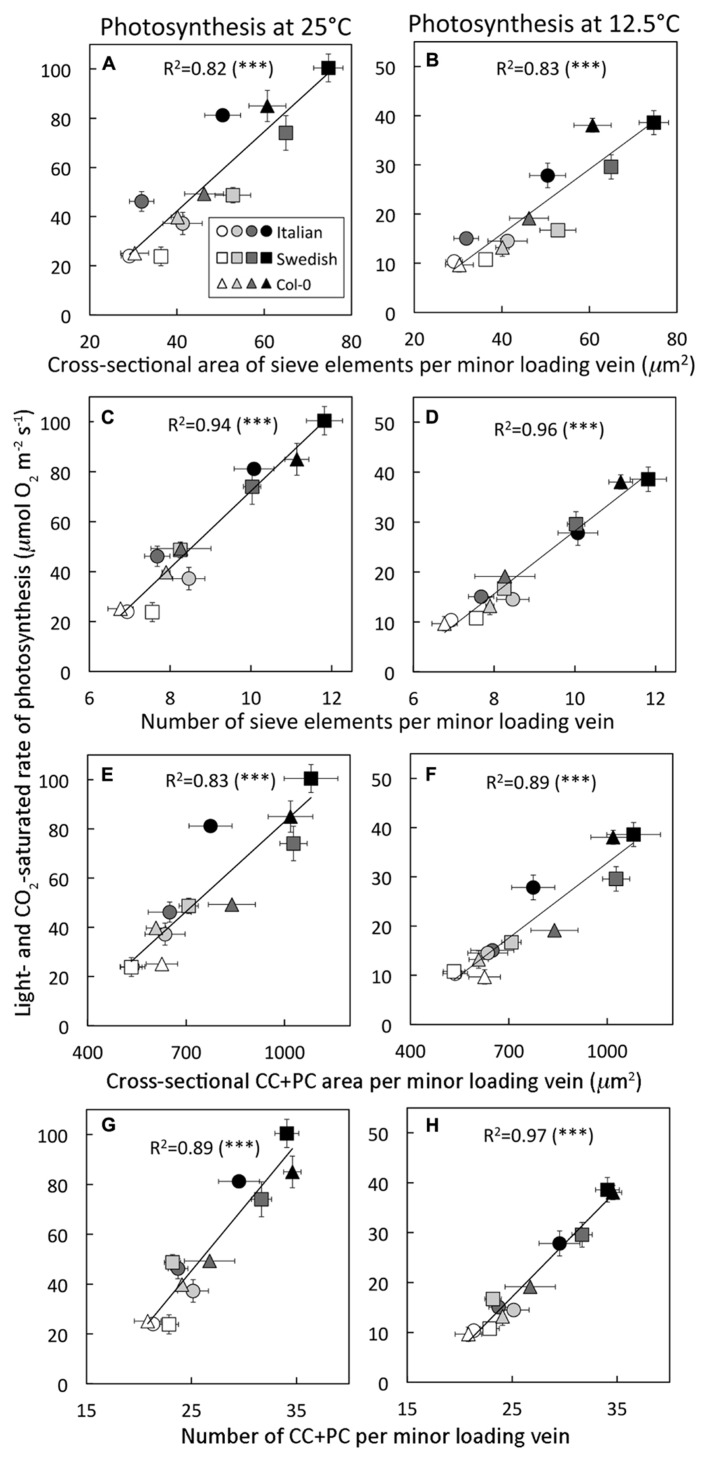
**Light- and CO _2_-saturated rate of oxygen evolution, determined at either 25^°^C or 12.5^ °^C, versus (A,B) the cross-sectional area of sieve elements per minor loading vein, (C,D) the number of sieve elements per minor loading vein, (E,F) the cross-sectional area of companion and phloem parenchyma cells (CC + PC) adjacent to sieve elements per minor loading vein, and **(G,H)** the number of companion and phloem parenchyma cells adjacent to sieve elements per minor loading vein for three *A. thaliana* ecotypes grown at two leaf temperatures and two PFDs.** *Arabidopsis thaliana* lines (Italian ecotype, circles; Col-0, triangles; Swedish ecotype, squares) were grown under four different conditions (24–26°C and 400 μmol photons m^-^^2^ s^-^^1^, open symbols; 24–26°C and 1000 μmol photons m^-^^2^ s^-^^1^, light gray symbols; 12–16°C and 400 μmol photons m^-^^2^ s^-^^1^, dark gray symbols; 12–16°C and 1000 μmol photons m^-^^2^ s^-^^1^, black symbols). Mean ± standard deviation shown for light- and CO_2_-saturated rates of photosynthetic oxygen evolution, and mean ± standard error of the mean shown for number and cross-sectional area of cells per minor loading vein (*n* = 4 plants). Linear regression lines are shown with the following equations: **(A)** *y* = 1.6*x* - 22.4, **(B)** *y* = 0.7*x* - 10.4, **(C)** *y* = 15.4*x *- 81.9, **(D)** *y* = 6.3*x* - 34.8, **(E)** *y* = 0.12*x* - 38.7, **(F)** *y* = 0.05*x* - 19.83, **(G)** *y* = 5.1*x* - 82.1, and **(H)** *y* = 2.1*x* - 36.4. All relationships were significant at ***P < 0.001.

Multiple features of the phloem component of minor loading veins turned out to be excellent predictors of a leaf’s light- and CO_2_-saturated rate of photosynthetic oxygen evolution (**Figure 4**), including cross-sectional area of sieve elements (**Figures 4A,B**), total number of sieve elements (**Figures 4C,D**), cross-sectional area of companion and phloem parenchyma cells (CC + PC; **Figures 4E,F**), and number of CC + PC (**Figures 4G,H**). **Figure 4** depicts significant linear relationships between the light- and CO_2_-saturated rate of photosynthetic oxygen evolution versus the latter phloem features in minor loading veins of all three ecotypes grown under four different conditions (two leaf temperatures and two PFDs) when photosynthesis was measured at either 25 or 12.5°C. These positive linear relationships were strongest for light- and CO_2_-saturated rates of photosynthetic oxygen evolution versus the *number* of either sieve elements (**Figures 4C,D**) or CC + PC (**Figures 4G,H**) per loading vein when compared to the *areas* of either sieve elements (**Figures 4A,B**) or CC + PC (**Figures 4E,F**).

**FIGURE 5:**
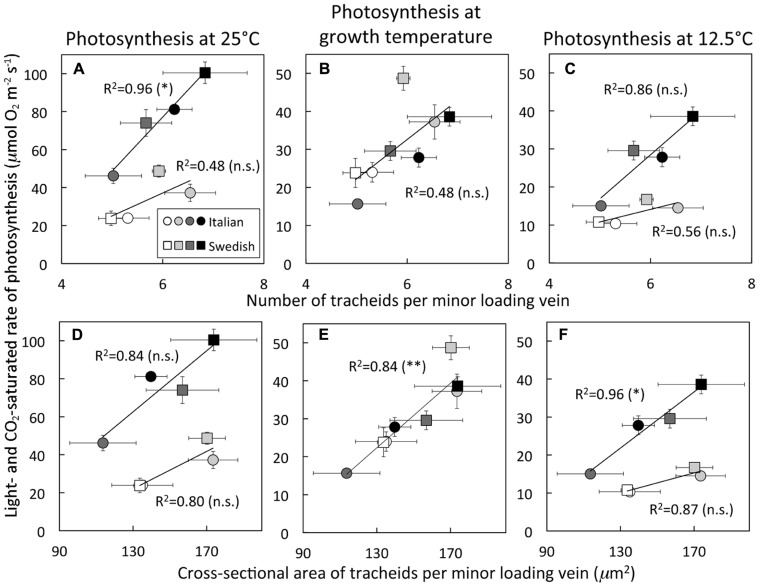
**Light- and CO _2_-saturated rate of oxygen evolution, determined at either 25 or 12.5^°^C, versus **(A–C)** the number of tracheids per minor loading vein or (D–F) the cross-sectional area of tracheids per minor loading vein for Italian (circles) and Swedish (squares) ecotypes of *A. thaliana* grown at two leaf temperatures and two PFDs.** Plants were grown under four different conditions (24–26°C and 400 μmol photons m^-^^2^ s^-^^1^, open symbols; 24–26°C and 1000 μmol photons m^-^^2^ s^-^^1^, light gray symbols; 12–16°C and 400 μmol photons m^-^^2^ s^-^^1^, dark gray symbols; 12–16°C and 1000 μmol photons m^-^^2^ s^-^^1^, black symbols). Mean ± standard deviation shown for light- and CO_2_-saturated rates of photosynthetic oxygen evolution, and mean ± standard error of the mean shown for number and cross-sectional area of tracheids per minor loading vein (*n* = 4 plants). Linear regression lines are shown with the following equations: **(A)** growth at 14°C line by *y* = 28.4*x* - 93.4 and growth at 25°C line by *y* = 12.0*x* - 35.1, **(B)** *y* = 10.5*x* - 30.4, **(C)** growth at 14°C line by *y* = 11.6*x* - 41.0 and growth at 25°C by *y* = 3.3*x* - 5.8, **(D)** growth at 14°C line by *y* = 0.8*x* - 41.9 and growth at 25°C by *y* = 0.5*x* - 42.1, **(E)** *y* = 0.4*x* - 34.0, and **(F)** growth at 14°C line by *y* = 0.4*x* - 26.2 and growth at 25°C line by *y* = 0.1*x* - 7.0. Level of significance (in parentheses) indicated as follows: *P < 0.05, **P < 0.01, and n.s., not statistically significant.

While the total cross-sectional area of the entire xylem per vein, unlike phloem area, was not significantly associated with light- and CO_2_-saturated rates of photosynthetic oxygen evolution (**Figure 1C**), the tracheid cells of the xylem did show some associations with photosynthesis. Light- and CO_2_-saturated rates of photosynthetic oxygen evolution versus tracheid number per vein (**Figures 5A–C**) or total cross-sectional tracheid area per vein (**Figures 5D–F**) for Italian and Swedish ecotypes also yielded linear relationships, several of which were significant, while most were not significant. In contrast to the relationships with the cells of the phloem (**Figure 4**), light- and CO_2_-saturated rates of photosynthetic oxygen evolution versus tracheids exhibited two *separate* linear relationships corresponding to the two different growth temperatures when photosynthesis was determined at either 25°C (**Figures 5A,D**) or 12.5°C (**Figures 5C,F**), but converged on a *single* linear relationship when photosynthesis was measured at the approximate respective *growth* temperatures (**Figures 5B,E**). Growth under higher PFD at a given temperature consistently resulted in greater numbers and a larger total cross-sectional tracheid area per minor loading vein (**Figure 5**). Furthermore, growth at higher temperature resulted in a greater number and a larger total cross-sectional tracheid area per minor loading vein in the Italian ecotype under a given growth light regime, but not in the Swedish ecotype (**Figure 5**). It is also important to note that foliar vein density was not significantly different among all three ecotypes under any of the growth conditions (mean ± standard deviation = 2.78 ± 0.25 mm vein length per mm^2^ leaf area, *n* = 48 plants; **Table 1**).

## DISCUSSION

We previously suggested that specialized architectural features of foliar phloem in plants (symplastic loaders) that load sugars into minor veins through plasmodesmata may provide a physical limitation to sugar export preventing full acclimation of photosynthesis in mature leaves acclimated to one condition and subsequently transferred to another (Amiard et al., 2005; Adams et al., 2007). Gifford and Evans (1981) and Wardlaw (1990) had previously speculated, based on the then-available evidence, that transport of photosynthetic products from sources to sinks was unlikely to be limiting. On the other hand, Ainsworth and Bush (2011) recently suggested that one might be able to enhance photosynthesis and plant productivity by increasing the capacity for exporting carbohydrates from leaves through the phloem.

The present report provides the first data linking photosynthesis to aspects of the basic phloem structure of leaves. The association reported here between a leaf’s light- and CO_2_-saturated rate of photosynthetic oxygen evolution and the proportion of minor loading veins devoted to phloem, and particularly the highly significant linear relationship between number, or total cross-sectional area, of these vein’s sugar-loading cells and sugar-transporting sieve elements versus their light- and CO_2_-saturated rates of photosynthetic oxygen evolution (**Figure 4**), strongly suggests that the capacity for exporting sugars from the leaf is a contributing determinant of a leaf’s maximally achievable rate of photosynthesis. It should, however, be noted that these data are correlative in nature, and that causality should be addressed in future studies.

The remarkable light- and CO_2_-saturated rate of 108 μmol O_2_m^-^^2^s^-^^1^ exhibited by the leaves of the *A. thaliana* ecotype from Sweden grown under high PFD and cool temperatures is several times higher than the photosynthetic rate of *A. thaliana* Col-0 leaves grown under high PFD and warm temperatures (close to 40 μmol O_2_ m^-^^2^ s^-^^1^ in the present study, **Figure 4**) and exceeds light- and CO_2_-saturated rates of photosynthetic oxygen evolution from herbaceous species growing in full sunlight in the winter (75 μmol O_2_ m^-^^2^ s^-^^1^for the winter annual spinach; Adams et al., 1995; 70–80 μ mol O_2_ m^-^^2^ s^-^^1^ for the biennial *Malva neglecta*; Adams et al., 2002). Our findings suggest that potential future efforts to overexpress sieve element and companion cell number (and thereby presumably sugar export capacity) may reveal that the upper limit of photosynthesis is considerably higher than currently assumed.

The linear associations between a leaf’s light- and CO_2_-saturated rate of photosynthetic oxygen evolution and number or cross-sectional area of tracheids per minor loading vein are consistent with previous studies emphasizing the transport and distribution of water to and within leaves in support of transpirational water loss during CO_2_ uptake through the stomata (Hubbard et al., 2001; Brodribb et al., 2005, 2010; Nardini et al., 2005; Sack and Holbrook, 2006; Boyce et al., 2009; Beerling and Franks, 2010; Brodribb and Feild, 2010; McKown et al., 2010; Blonder et al., 2011; Walls, 2011). Increasing tracheid numbers, and consequently an increasing total cross-sectional tracheid area, per minor loading vein exhibited a *single* linear relationship with increased light- and CO_2_-saturated rates of photosynthesis when determined at the respective growth temperature (25 or 12.5°C) of each set of leaves, but *two separate* relationships when determined at one or the other temperature. This finding suggests that the architecture of water-transporting xylem cells is adjusted specifically for the temperature and PFD under which leaves develop, presumably to provide for physical delivery of water (along a water potential gradient) at a rate matching the demand of transpirational water loss to the specific environmental conditions under which plants were growing.

On the other hand, presumably as the integrated coordination of two processes that *both* rely on proteins (enzymes of the Calvin cycle in photosynthesis and transport proteins in phloem loading) subject to decreased activity with decreases in temperature, light- and CO_2_-saturated rates of photosynthetic oxygen evolution increased linearly with the number or cross-sectional area of phloem cells among all ecotypes and growth conditions when photosynthesis was determined at a single temperature. At any given temperature, the light- and CO_2_-saturated photosynthesis rate of leaves that developed at lower temperature was thus greater than that of leaves that developed at warm temperature, such that the overall effect allowed leaves to maintain a similar light- and CO_2_-saturated photosynthesis rate at the lower growth temperature as leaves growing under the warmer growth temperature. In other words, leaves growing at low temperature upregulated photosynthesis, as well as their apparent capacity to export products of photosynthesis through more phloem cells, to maintain a rate of photosynthesis similar to that of leaves growing at warm temperature. For *A. thaliana*, which is an apoplastic loader (Haritatos et al., 2000), the increased number of companion and phloem parenchyma cells in minor veins of leaves that developed under lower temperature and higher PFD presumably provide for an increased cell membrane area for greater numbers of transport proteins (sucrose-H^+^ co-transporters and adenosine triphosphatases) to drive the loading of sugars into the phloem. Concomitantly, the greater numbers of sieve elements per vein provide a greater cross-sectional area through which sugars can be transported, which may be important to accommodate greater sugar production resulting from higher rates of photosynthesis and/or a more viscose phloem sap that could arise from decreasing temperature (Cohu et al., 2013). Plants acclimated to lower temperature, however, presumably incur higher costs in terms of the nitrogen and energy invested into greater numbers of cells and photosynthetic and transport proteins.

Exploration of differences among the three *A. thaliana* ecotypes revealed that the Swedish ecotype not only exhibited the greatest number and combined size of sieve elements, but also showed the most sensitive response to variation of growth conditions, with Col-0 (presumed origin in Germany) showing an intermediate response, and the Italian ecotype showing the least ready response to temperature (**Figure 3**). This difference among ecotypes prepares the ground for future molecular approaches to identify the gene(s) responsible for increased augmentation of the phloem in minor loading veins and high photosynthetic rates of the Swedish versus the Italian ecotype. Furthermore, the observed differences between the Swedish and Italian ecotypes may also be relevant to the performance of the two subjected to reciprocal transplants. Both exhibited lower survival and lower reproductive fitness in comparison to the local populations when transplanted to the other’s habitat (Ågren and Schemske, 2012). In the case of the Italian ecotype grown in Sweden, reduced performance might be due to an inability to increase the numbers of phloem cells to the same level as observed in the Swedish ecotype to facilitate enhanced rates of sugar export and transport and of photosynthesis at the lower temperatures prevailing in Sweden. On the other hand, the relatively poorer performance of the Swedish ecotype relative to the Italian ecotype when growing in Italy may be due to differences between the two in the response of the xylem to growth temperature. Minor veins of the Italian ecotype possessed a greater number, as well as a greater total cross-sectional area, of tracheids in leaves that developed under warm compared to cool temperatures. Such a response may provide for a greater flux of water to meet the transpirational demands of plants experiencing warmer temperatures during growth, and thus would be more relevant for plants that grow in Italy (warmer and drier atmosphere) compared to Sweden. The minor vein tracheids of the Swedish ecotype exhibited the opposite response to growth temperature, with a greater number and cross-sectional area present in leaves that developed under cool compared to warm temperature. Such a response could be maladaptive to growth in the warmer and drier climate of Italy, but may be critical to complementing the greater number of phloem cells in the foliar minor veins of the Swedish ecotype when leaves develop at lower temperature. Nikinmaa et al. (2013) recently reported that the water potential gradient from xylem to phloem exceeds that between xylem and transpirational water loss. It is thus conceivable that, for the Swedish ecotype that evolved in a cooler and moister climate, it is advantageous to link development of more tracheids to the development of more phloem in response to growth at cool temperatures rather than to warm temperatures rarely experienced by this winter annual in a high-latitude environment.

Removal of sinks (like developing fruit or developing leaves) as destinations for sugars produced by photosynthetically active source leaves results in decreased photosynthesis rates of source leaves (Mondal et al., 1978; Myers et al., 1999; Wünsche et al., 2005; Duan et al., 2008). In addition, downregulation of components of the photosynthetic apparatus, due to feedback inhibition by products of photosynthesis accumulating in the leaf, was elegantly demonstrated through cold-girdling (lowering the temperature of the phloem to slow the transport of sugars out of the leaf) of individual leaf petioles (Krapp et al., 1993; Krapp and Stitt, 1995). Physical removal of the phloem (girdling) of whole branches likewise resulted in strong downregulation of photosynthesis in leaves of those branches (Myers et al., 1999; Urban and Alphonsout, 2007). It can be concluded that sustained increases in photosynthesis of mature leaves must involve effective carbohydrate removal from the source leaves. Increased investment by the plant in expensive-to-maintain photosynthetic machinery should thus only be expected if sufficient sugar-export infrastructure is available to distribute the resulting increased sugar production.

We therefore propose that number (and total cross-sectional area) of the sieve elements of a leaf’s loading veins can represent a limitation to sugar export from leaves that represses photosynthetic genes and limits photosynthesis. It is important to note that additional foliar features co-vary with photosynthetic rate, including the stomatal pores through which CO_2_ gains access to the chloroplasts (Farquhar and Sharkey, 1982; Medrano et al., 2002), hydraulic conductivity of the plant’s water transport system (Hubbard et al., 2001; Brodribb et al., 2005, 2010; Nardini et al., 2005; Sack and Holbrook, 2006), leaf thickness and dry mass per area (Wright et al., 2004; Terashima et al., 2006; Dumlao et al., 2012), total chloroplast area exposed to leaf internal air spaces (Oguchi et al., 2005; Terashima et al., 2006), and leaf nitrogen content (Küppers et al., 1995; Hikosaka, 2004; Wright et al., 2004). Nonetheless, our findings suggest that environmental cues can trigger the production of greater numbers of phloem cells in minor loading veins – as an increase in foliar vascular infrastructure presumably required for maximal sugar distribution and to allow up-regulation of photosynthetic sugar production. The latter finding has implications for the fundamental understanding of what governs leaf photosynthetic activity. The number of phloem cells in minor loading veins may play a major role in setting the capacity of the distribution route for sugars produced in photosynthesis to the plant’s sugar-storing and sugar-consuming sinks. If this anatomical feature were to represent a bottleneck, its manipulation might allow photosynthesis to be increased to unprecedented levels. Furthermore, as atmospheric carbon dioxide levels continue to rise, the ability to increase the flux of sugars and other reduced carbon compounds out of the leaves to the rest of the plant may become critical to preventing the downregulation of photosynthesis that can result from the accumulation of sugars in leaves in response to elevated CO_2_ levels (van Oosten and Besford, 1996; Makino and Mae, 1999; Moore et al., 1999; Paul and Foyer, 2001; Bloom, 2006). One important aspect of the present findings thus lies in the identification of a potential target to increase photosynthesis via selection, breeding, and/or engineering of crop varieties with the greatest propensity for enhancement of foliar sugar-export. Concomitant assessment of foliar minor loading vein phloem features and light- and CO_2_-saturated rates of photosynthetic oxygen evolution of different species (Adams et al., 2013) can also serve to further test the generality of the fundamental relationship described here.

## Conflict of Interest Statement

The authors declare that the research was conducted in the absence of any commercial or financial relationships that could be construed as a potential conflict of interest.

## Footnotes

1 http://www.arabidopsis.org/

2 http://imagej.nih.gov/ij/
